# Effective inclusion practices for neurodiverse children and adolescents in informal STEM learning: a systematic review protocol

**DOI:** 10.1186/s13643-023-02278-2

**Published:** 2023-07-01

**Authors:** Ronda J. Jenson, Michele S Lee, Arden D. Day, Amy E. Hughes, Emma E. Maroushek, Kelly D. Roberts

**Affiliations:** 1 Psychological Sciences, Northern Arizona University, Flagstaff, USA; 2 Institute for Human Development, Northern Arizona University, Flagstaff, USA; 3 Cline Library, Northern Arizona University, Flagstaff, USA

**Keywords:** Neurodiversity, Informal learning, STEM, Children, Adolescents, Systematic review

## Abstract

**Background:**

Informal learning experiences in science, technology, engineering, and math (STEM) can enhance STEM learning that occurs in formal educational settings and curricula as well as generate enthusiasm for considering STEM careers. The aim of this systematic review is to focus on the experiences of neurodiverse students in informal STEM learning. Neurodiversity is a subgroup of neurodevelopmental conditions, such as autism, attention deficit disorder, dyslexia, dyspraxia, and other neurological conditions. The neurodiversity movement regards these conditions as natural forms of human variation, as opposed to dysfunction, and recognizes that neurodiverse individuals possess many strengths relevant to STEM fields.

**Methods:**

The authors will systematically search electronic databases for relevant research and evaluation articles addressing informal STEM learning for K-12 children and youth with neurodiverse conditions. Seven databases and content-relevant websites (e.g., informalscience.org) will be searched using a predetermined search strategy and retrieved articles will be screened by two members of the research team. Data synthesis will include meta-synthesis techniques, depending on the designs of the studies.

**Discussion:**

The synthesis of the findings resulting from various research and evaluation designs, across the K-12 age span, and across various informal STEM learning contexts, will lead to depth and breadth of understanding of ways to improve informal STEM learning programs for neurodiverse children and youth. The identification of informal STEM learning program components and contexts shown to yield positive results will provide specific recommendations for improving inclusiveness, accessibility, and STEM learning for neurodiverse children and youth.

**Trial registration:**

The current study has been registered in PROSPERO. Registration number: CRD42021278618.

## Background

Informal STEM learning is “learning in science, technology, engineering, and math that takes place across a multitude of settings and experiences outside of the formal classroom” [1]. Informal learning environments are, in principle, accessible to all learners, and evidence suggests that they have potential for creating unique opportunities for learners from underrepresented populations [2]. Informal learning environments can be categorized into three major settings: everyday experiences, designed settings, and programmed settings [3]. In each of these settings, informal learning experiences are typically self-directed, where the learner has the freedom to make choices to guide their own learning, pursue interests, and, as a result, take responsibility for their own learning [4, 5]. Self-directed, informal learning environments create more accessible learning opportunities by allowing students to start the learning process at whatever point best meets their own needs and allowing them to go at their own pace. Through self-directed informal learning, children and youth, both with and without disabilities, can (a) access hands-on learning in individual and group settings, (b) explore the STEM environment freely without the pressures associated with testing or making mistakes, (c) take additional time or repetitions as needed to fully explore and learn, and (d) engage in real-world and culturally relevant STEM problems and solutions [6]. This self-directed pedagogical approach promotes self-determination [4, 7], fosters motivation to learn [8–11], and generates interest in STEM [3, 12, 13].

Participation in informal STEM environments contributes to academic aspirations, as well as perceived competence, interest, confidence, and engagement in science and math concepts [3, 4, 14–16]. When designed to be inclusive for learners with and without disabilities, informal science learning experiences empower science learners by generating enthusiasm for science and fostering equitable learning experiences [6]. Inclusive learning settings can increase interest in and enthusiasm for STEM and may result in increased career outcome expectancy and retention of students pursuing STEM careers, thus diversifying the workforce [17–19].

### Defining neurodiversity and implications for STEM learning

While there are various views on disability, the understanding of disability as a social construct is the most widely accepted. As opposed to a medical view, in which the disabling condition is construed as a problem to be fixed, the social model of disability considers variations in ability to be a natural human difference [20]. By acknowledging this human variance, environments, policies, and programs can be designed to accommodate and welcome diversity. The neurodiversity movement is based on “cerebral pluralism, the idea that each brain is different, some more different than others” [21]. The term “neurodiversity” was coined by a sociologist in 1990 to describe conditions such as autism spectrum disorder, attention deficit hyperactivity disorder (ADHD), dyslexia, dyspraxia, and other neurological conditions characterized as less-typical cognitive variation [22]. While neurodiversity is not a clinical term, it does encompass some clinical diagnoses such as autism spectrum disorder. This study’s use of the term neurodiversity was guided by the project’s funding source, the National Science Foundation (NSF) who relies on field-determined definitions which can be found by examining the portfolio of awards that include elements focused on neurodiversity. Students with neurodivergent conditions often face hurdles associated with (a) managing day-to-day activities, (b) adapting to changes in routines, (c) navigating social interactions, (d) sensory demands, and (e) barriers posed by disability-related bias or social stigma [23–26].

Studies about informal STEM learning settings and neurodiverse learners have explored the experiences of elementary-age students with autism in a museum program [27, 28]; after-school robotics programs for youth with autism and intellectual disabilities [29]; the use of tablets as technologies in robotic education for youth with neurodevelopmental disorders, including motor disabilities and autism [30]; embedding the use of mobile technologies in informal learning and implications for youth with dyslexia [31]; and the organization of an inclusive STEM camp [32]. Additionally, recent research has studied the outcomes of informal learning experiences for specific neurodiverse populations with respect to increased engagement, interest, or cognitive skills [33, 34]. These studies demonstrate that informal STEM learning can serve children with differing types of disability conditions from early childhood through adolescence in varying types of learning settings. Informal STEM environments often provide children with opportunities to explore STEM topics that they are interested in [29], leading to increased confidence in their ability to pursue a STEM career [30]. As previous research has demonstrated, increased confidence in science and math skills is correlated with STEM identity and interest in STEM careers [16].﻿ Informal learning settings for the proposed project are out-of-school programs, such as, an after-school program or summer camp.

**Fig. 1:**
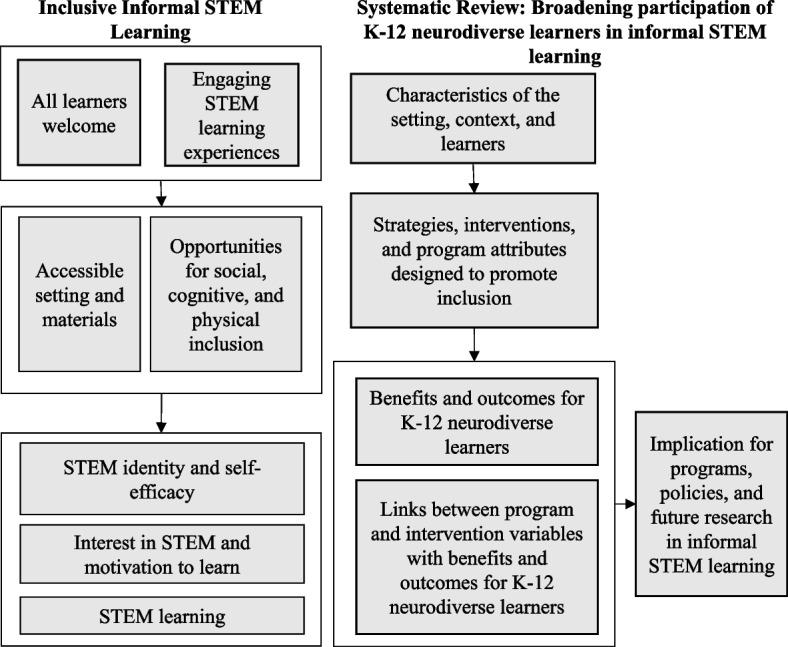
Theoretical framework

Currently, there is no comprehensive review that synthesizes literature on informal STEM experiences and outcomes for neurodiverse K-12 children and youth. The purpose of this systematic review is to synthesize the evidence of informal learning programs for neurodiverse children and adolescents in the United States. The proposed project will review studies from the United States only. Justification for inclusion of studies that took place only in the United States is to be consistent with the scope of informal STEM learning programs funded by the National Science Foundation. Figure 1 shows the theoretical framework guiding the proposed systematic literature review. Inclusive informal STEM learning is welcoming and accessible to all learners while the learning environment and experiences offer opportunities for inclusion. The outcome measures for this project include programmatic elements (teaching and learning variables) in informal STEM learning settings that facilitate inclusion of neurodiverse K-12 STEM learners and benefits to neurodiverse K-12 STEM learners (e.g., STEM interest, STEM identity, STEM self-efficacy, and STEM learning). The results and benefits of engaging in the inclusive opportunities are (1) increased STEM identity and self-efficacy [35], (2) increased interest in STEM and motivation to learn [17], and (3) increased STEM learning [36]. This systematic review  used the proposed theoretical framework  to conceptulize the research questions.

## Methods/design

### Research questions

RQ1. What program elements (teaching and learning variables) in informal STEM learning settings facilitate inclusion of neurodiverse K-12 STEM learners?

Sub-RQ1a: What are the overlapping and discrete characteristics of the program elements that facilitate social, cognitive, and physical inclusion?

Sub-RQ1b: In what ways do the program elements that facilitate inclusion vary by informal STEM learning setting?

RQ2: What program elements (teaching and learning variables) in informal STEM learning settings are correlated with benefits for neurodiverse K-12 STEM learners?

Sub-RQ2a: What are the overlapping and discrete characteristics of the program elements that correlate with increased STEM identity, self-efficacy, interest in STEM, or STEM learning?

Sub-RQ2b: In what ways do the program elements that correlate with positive results for students vary by informal STEM learning setting?

### Study design

This project will use a comprehensive definition of “systematic review,” which is “a process of systematically bringing together the results of any research, including qualitative or mixed methods research studies” [37]. In order to uncover what works, what it looks like, how it works, and for whom it works, this systematic review will encompass the following types of studies: (a) research and evaluation; (b) experimental and quasi-experimental designs; (c) quantitative, qualitative, and mixed methods; and (d) implementation studies [38].

### Search strategy

Seven databases will be searched: Academic Search Complete, Cochrane Library, Education Full Text, ERIC, PsycINFO, ScienceDirect, and Web of Science. Electronic databases were selected based upon their inclusion and coverage of relevant disciplinary content, specifically disability studies, teaching and learning, education, and informal learning environments. Each database will be systematically searched using customized search strategies which leverage controlled vocabulary specific to each resource, and universal key teams that will be searched across all resources. Main subject terms or thesaurus terms will be searched as keywords. Additional synonyms and related keywords will be included and were derived from the literature. All terms will be  combined using the OR Boolean operator, to create an exhaustive search using several keywords.

Searching within Google Scholar and the informal science online resource managed by the Center for Advancement of Informal Science Education (CAISE) will supplement the process, capturing any relevant grey literature. Additional grey literature sources will include relevant education and informal science repositories. Justification for searching literature that was produced from 2008 and on was based on a National Research Council report that indicated the literature on STEM informal learning for persons with disabilities was ‘thin’ prior to 2008 and since then the amount of literature has grown [39].

**Fig. 2:**
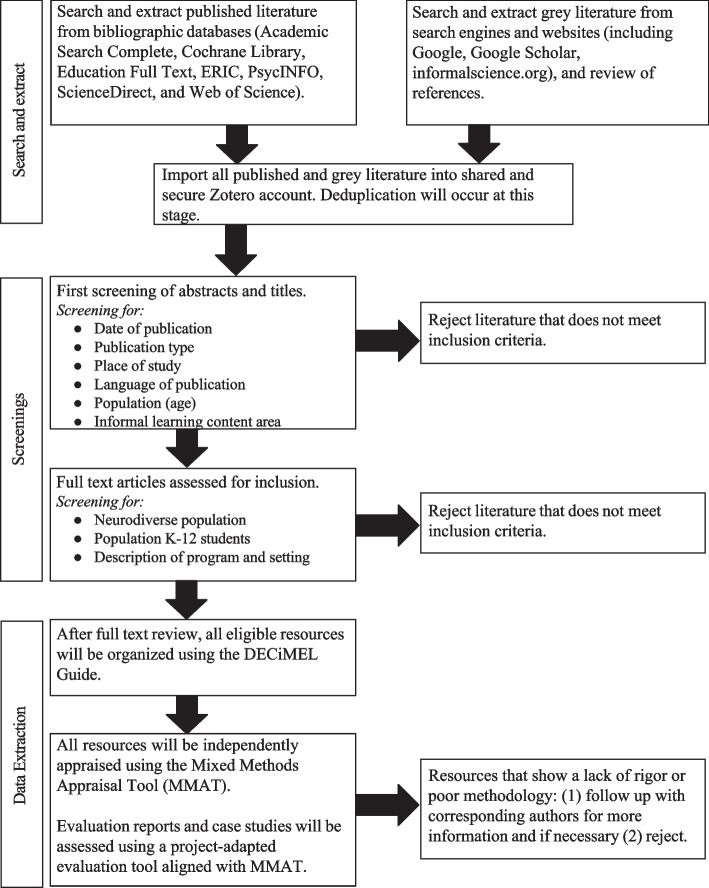
Visual representation of the search strategy, screening, and data extraction

Databases will be searched separately. Article citations and abstracts will be exported into a corresponding folder within the team’s shared citation management account. Open access journals not covered in the databases will be hand searched. Additional articles, reports, and case studies will be extracted from relevant websites (e.g., informalscience.org) and repositories, and imported into the account. Once the information has been collected, duplicate records will be identified and merged. See Fig. 2 for a visual representation of the search strategy, screening, data extraction, and quality appraisal approach.

### Screening

As shown in Fig. 2, the team will conduct two screenings using pre-established inclusion and exclusion criteria prior to a full-text review. In the first screening, two reviewers will independently screen the article title and abstract for inclusion criteria. If one reviewer indicates an article as relevant at the initial screening phase, the article will proceed to the second screening to ensure inclusivity. In the second screening, two reviewers will screen the full text of articles to ensure the second inclusion criterion is met. Reviewers will establish inter-rater agreement through a process of trial screening of articles and calibration of the inclusion/exclusion criteria. Once a Kappa coefficient of inter-rater agreement of 0.8 is achieved, the reviewers will proceed to screen the articles. As shown in Table 1, articles will be screened for the following criteria.

**Table 1:** Inclusion criteria using SPIDER [40]

Sample	Children and adolescents between ages five and 19, or K-12 students who are neurodiverse or identify as having a neurodevelopment disorder
Phenomenon of Interest	Describes an informal STEM learning experience
Design	Any quantitative design using quantitative methods of data collection and analysis; any qualitative design using qualitative methods of data collection and analysis; quantitative and qualitative components of mixed method studies
Evaluation	Programmatic elements (teaching and learning variables) in informal STEM learning settings that facilitate inclusion of neurodiverse K-12 STEM learners and benefit neurodiverse K-12 STEM learners (e.g., STEM interest, STEM identity, STEM self-efficacy, and STEM learning)
Research type	Quantitative, qualitative, or mixed method study designs

In the case of missing data, a research team member will contact the corresponding author for unreported data or additional details. If the corresponding author does not respond, the research team will not include the study. Additionally, if the study details do not allow results to be disaggregated for children/adolescents with neurodiverse conditions or by age the study will not be included. It is expected that some data may include parent and/or program leader perspectives of child/adolescent experiences in STEM learning in lieu of direct assessment of child/adolescent learning.

### Data extraction

The full text of all articles meeting inclusion criteria will be reviewed. Relevant information will be extracted from the articles using the data extraction for complex meta-analysis (DECiMAL) guide when studies involve quantitative results [41]. Extracted data will include the type of study, study design, and methodology. Information about the populations will also be extracted and will include number of participants, location, program activities, and neurodiverse condition(s) described. Extracting qualitative data will involve content review and coding for themes [42]. Researchers will maintain a coding tree with descriptors to ensure consistent use of labels. NVivo will be used for organizing content by codes. The research team will develop a codebook containing all extracted elements accompanied by clear definitions.

### Quality appraisal

The quality of included studies will be independently appraised by two reviewers using the Mixed Methods Appraisal Tool (MMAT) [43, 44]. The MMAT is a 21-item checklist used to rate the quality of quantitative, qualitative, and mixed methods studies selected for review. If discrepancies arise, a third reviewer will complete an assessment for consensus. Recognizing that the MMAT may not be applicable to grey literature (i.e., case studies), the research team will use a project-adapted evaluation tool aligned with the MMAT. To ensure the consistency of MMAT and the project-adapted MMAT, calibration exercises will be conducted using a sample of 15 papers to assess for reviewer consistency. Studies rated as lower quality using the MMAT will not be excluded; however, the study quality will be reported when results are synthesized and published. While inclusion of lower quality studies seems counterproductive, research comparing systematic review results shows the potential for bias when studies not meeting the highest standards of rigor are not included [45].

### Synthesis

Overall summarized article results will be reported using the Preferred Reporting Items for Systematic Reviews and Meta-Analyses (PRISMA) flow diagram [46]. The characteristics of studied interventions, contexts, and implementation will be reviewed to determine the most appropriate method of synthesis, to include the effects, relationships, similarities and differences, descriptions of implementation, and relevance to contexts and groups of people. If able to, comparisons will be made across different neurodiversity types and different tyles of informal learning interventions. Depending on the types of outcome variables collected, this will determine our ability to synthesize content. For example, if learning experiences are only noted in the literature, we will be limited in our ability to conduct quantitative analyses.

For quantitative studies, if the research includes a randomized control trial (RCT) or quasi-experimental study these interventions will be described. Results from studies with similar interventions and/or outcome measures will be examined via a meta-analysis to determine intervention effects on programmatic elements (teaching and learning variables) in informal STEM learning settings that facilitate inclusion of neurodiverse K-12 STEM learners and benefits to neurodiverse K-12 STEM learners (e.g., STEM interest, STEM identity, STEM self-efficacy, and STEM learning). Because broadening participation in informal STEM learning is an emerging area of research, a pair-wise meta-analysis may be unlikely. In that case, the research team will use alternative appropriate methods (e.g., network meta-analysis, Bayesian meta-analysis). If sufficient data do not exist, findings will be reported in a narrative synthesis organized by outcome variables.

For qualitative studies, meta-synthesis will occur using fuzzy cognitive mapping (FCM) after data is coded and organized into descriptive theme groups and analytical theme groups to answer the research questions. If possible, FCM will be used as a form of content analysis to examine the compiled results from a systems level perspective [47]. FCMs are frequently used to model interdependence between concepts through a graphical representation of causal relationships. The summary FCM will be generated by examining “Results” sections from the qualitative articles for antecedent-consequent linguistic statements (e.g., “if… then…”) that connect specific intervention characteristics to specific study outcomes. Only connections that are supported by at least two articles will be included in the final FCM. For mixed method studies, data synthesis will depend on the type of mixed method design (e.g., convergent vs. sequential synthesis design).

## Discussion

This systematic review will add to the knowledge base on effective STEM instruction for students with disabilities by focusing on informal STEM learning environments and the benefits experienced by neurodiverse learners as listed in Fig. 1. Identification of the program elements in informal STEM learning settings that facilitate inclusion of and learning for neurodiverse K-12 STEM learners is important for increasing access to STEM learning, improving STEM learning, STEM-interest and building self-efficacy as a STEM learner.

The results will be useful for informal STEM program leaders, STEM educators, and STEM education researchers. Informal STEM program leaders will be able to use results and compiled recommendations as a resource containing concrete descriptions and reflections regarding structures and contexts for accessible informal STEM learning opportunities and generation of ideas for making improvements in their own programs. STEM educators will be able to use the results to inform the focus and delivery of STEM instruction through a lens of including students with disabilities. STEM education researchers will be able to build upon the results by developing and studying innovative interventions and conducting research to address gaps revealed through the systematic review.

Given that the literature on this subject is in an early state of research, one potential limitation could be inconsistency in intervention details among studies. Diverse outcome measures in assessing informal STEM learning may limit our ability to combine results from different studies, which impacts our ability to disentangle the magnitude of effects of the informal STEM intervention. There also may be insufficient statistical power in the smaller studies. Given the potential limitations, it also may be necessary to consider how quality appraisal is evaluated. The research team plans to use the MMAT but will need to monitor the appropriateness of this tool regarding the literature pulled. The results of this systematic review will be reported in a peer-reviewed journal. Any amendments made to this protocol during the review will be reported in PROSPERO and reported in the final manuscript.
